# Physical Resilience After Hip Fracture: Unpacking the Roles of Resistance and Recovery

**DOI:** 10.1093/geroni/igaf122.1059

**Published:** 2025-12-31

**Authors:** Chenkai Wu, Jianhong Xu, Yanxin Wang, Qian-Li Xue, Graciela Muniz-Terrera

**Affiliations:** Duke Kunshan University, Kunshan, Jiangsu, China (People’s Republic); Duke Kunshan University, Kunshan, Jiangsu, China (People’s Republic); Duke Kunshan University, Kunshan, Jiangsu, China (People’s Republic); Johns Hopkins University, Baltimore, Maryland, United States; University of Edinburgh, Edinburgh, Scotland, United Kingdom

## Abstract

Physical resilience—the ability to withstand, recover, or adapt after a stressor— is critical in older adults, particularly after events such as hip fractures. We conceptualize physical resilience to comprise two distinct but related components: resistance and recovery. These components were used to evaluate how individuals respond to the stress of a hip fracture, both in terms of their immediate impact and their ability to regain health over time. Using data from nearly 5,000 hip fracture participants, we used linear mixed-effects models to characterize a composite health status, derived from electronic health record diagnoses, one year before and after hip fracture for each individual. Resistance was assessed as the abrupt change in composite health status observed immediately after the fracture, while recovery represented the subsequent rate of change. We used Cox and Fine-Gray models to identify the associations of resistance and recovery with all-cause and cause-specific mortality, respectively. We observed a significant and sharp decline in the composite health measure at the time of hip fracture. Following the fracture, the rate of decline markedly accelerated when compared to the pre-fracture period. Lower resistance was associated with a 2.6-fold increase in mortality (95%CI=2.22-3.04), and slower recovery was related to a more modest, yet significant, increase in mortality (HR = 1.18, 95%CI=1.03-1.36). Low resistance was also associated with an increase in the risk of neurodegenerative mortality (sub-distribution HR = 5.78, 95%CI=3.73-8.95). Physical resilience is a key determinant of post-hip fracture outcomes. Identifying individuals with lower resilience may improve clinical interventions to enhance recovery and reduce mortality.

